# Renal cell tumors convert natural killer cells to a proangiogenic phenotype

**DOI:** 10.18632/oncotarget.27654

**Published:** 2020-06-30

**Authors:** Yue Guan, Christopher B. Chambers, Taylor Tabatabai, Ha Hatley, Kristin R. Delfino, Kathy Robinson, Shaheen R. Alanee, Sophia Ran, Donald S. Torry, Andrew Wilber

**Affiliations:** ^1^Department of Medical Microbiology, Immunology and Cell Biology, Southern Illinois University School of Medicine, Springfield, IL 62702, USA; ^2^Department of Surgery, Division of Urology, Southern Illinois University School of Medicine, Springfield, IL 62702, USA; ^3^Center for Clinical Research, Southern Illinois University School of Medicine, Springfield, IL 62702, USA; ^4^Department of Internal Medicine, Southern Illinois University School of Medicine, Springfield, IL 62702, USA; ^5^Simmons Cancer Institute, Springfield, IL 62702, USA

**Keywords:** kidney cancer, innate immunity, natural killer cells, immunosuppression, angiogenesis

## Abstract

Natural killer (NK) cells are classically associated with immune surveillance and destruction of tumor cells. Inconsistent with this function, NK cells are found in advanced human tumors including renal cell carcinoma (RCC). NK cells with non-classical phenotypes (CD56^+^CD16^dim/neg^; termed decidua NK (dNK) cells) accumulate at the maternal-fetal interface during embryo implantation. These dNK cells are poorly cytotoxic, proangiogenic, and facilitate placenta development. As similarities between embryo implantation and tumor growth exist, we tested the hypothesis that an analogous shift in NK cell phenotype and function occurs in RCC tumors. Our results show that peripheral NK (pNK) cells of RCC patients were uniformly CD56^+^CD16^bright^, but lacked full cytotoxic ability. By comparison, RCC tumor-infiltrated NK (TiNK) cells were significantly enriched for CD56^+^CD16^dim-neg^ cells, a phenotype of dNK cells. Gene expression analysis revealed that angiogenic and inflammatory genes were significantly increased for RCC TiNK versus RCC pNK populations, with enrichment of genes in the hypoxia inducible factor (HIF) 1α pathway. Consistent with this finding, NK cells cultured under hypoxia demonstrated limited cytotoxicity capacity, but augmented production of vascular endothelial growth factor (VEGF). Finally, comparison of gene expression data for RCC TiNK and dNK cells revealed a shared transcriptional signature of genes with known roles in angiogenesis and immunosuppression. These studies confirm conversion of pNK cells to a dNK-like phenotype in RCC tumors. These characteristics are conceivably beneficial for placentation, but likely exploited to support early tumor growth and promote metastasis.

## INTRODUCTION

In humans, natural killer (NK) cells make up 5–20% of the nucleated cells in the peripheral blood, and are identified as CD3^neg^ lymphocytes that express CD56 with or without CD16. These two subtypes of NK cells demonstrate distinct differences in phenotype and function. Most (90–95%) peripheral blood NK (pNK) cells are CD56^+^CD16^bright^ and exhibit efficient cytotoxic responses. A small percentage (5–10%) of pNK cells are CD56^+^CD16^dim/neg^ with little cytotoxic function and enhanced proinflammatory cytokine production, including gamma interferon (IFNγ), tumor necrosis factor alpha (TNFα), and granulocyte macrophage-colony stimulating factor (GM-CSF) [1]. Tumor-infiltrated NK (TiNK) cells have impaired tumor cytotoxicity and increased proinflammatory cytokine production [2, 3]. However, less is known regarding the proangiogenic properties of TiNK cells or the mechanisms driving functional conversion [4].

The predominant lymphocyte population in the decidua during early placentation is NK cells. These decidua NK (dNK) cells comprise 75% of lymphocytes at the implantation site are phenotypically CD56^+^CD16^neg^ and demonstrate limited to no cytotoxicity capacity [5–8]. Human dNK cells secrete angiogenic molecules, particularly placenta growth factor (PGF) and vascular endothelial growth factor A (VEGFA) [5], angiopoietins (ANG), and transforming growth factor-beta (TGFβ) [6]. Accumulation of dNK cells correlates with the formation of blood and lymphatic vessels, endometrial edema and vasodilation leading to increased uterine artery blood flow [7, 8]. Thus, CD56^+^CD16^neg^ NK cells could have significant, yet under-appreciated, roles in promoting angiogenesis in a number of pathological situations, including tumor growth and metastasis.

Renal cell carcinoma (RCC) is a major health issue with ~25% mortality rate at diagnosis. This is attributed to 40% of patients presenting with or developing metastatic disease and suboptimal efficacy of available treatments such as chemotherapy and radiation [9, 10]. Survival improves following surgery combined with cytokine therapy, but response rates have not exceeded 20% [11]. Suppression of tumor immunity by RCC likely imposes functional limitations on the effectiveness of immunotherapy [12]. Infiltration of NK cells at RCC sites is observed and these TiNK cells have limited cytotoxic potential and express differential repertoires of activating and inhibitory receptors [13–15]. The proangiogenic roles of NK cells in RCC and other tissue-specific cancers have not been investigated, and little has been reported about mechanisms responsible for the functional conversion of NK cells in the tumor environment.

Given the fundamental role of NK cells in tumor immunity, identifying the mechanisms by which tumors alter NK cell function may prove critical for controlling tumor growth. We investigated the extent to which RCC tumors could alter the phenotype and function of NK cells in the peripheral blood and tumor tissue of newly-diagnosed patients. We found that NK cells isolated from human RCC tumors are phenotypically different from matched pNK cells. Molecular characterization of RCC TiNK versus pNK revealed up-regulation of angiogenic and inflammatory genes, many of which were enriched in the hypoxia inducible factor (HIF) 1α pathway. In line with this finding, NK cells cultured under hypoxia demonstrated increased production of the angiogenic molecule VEGFA and reduced cytotoxic potential. Finally, comparison of upregulated genes to results from a published microarray for bona fide dNK cells [5] identified a shared genetic signature consisting of genes with known roles in vascularization and immunosuppression. These collective findings confirm that RCC tumors are able to alter the classical characteristics of NK cells towards a dNK-like program. While these characteristics are beneficial for placentation, they may be exploited to support RCC growth and metastasis.

## RESULTS

### Peripheral blood NK cells of RCC patients have reduced cytotoxic activity

**Figure 1:**
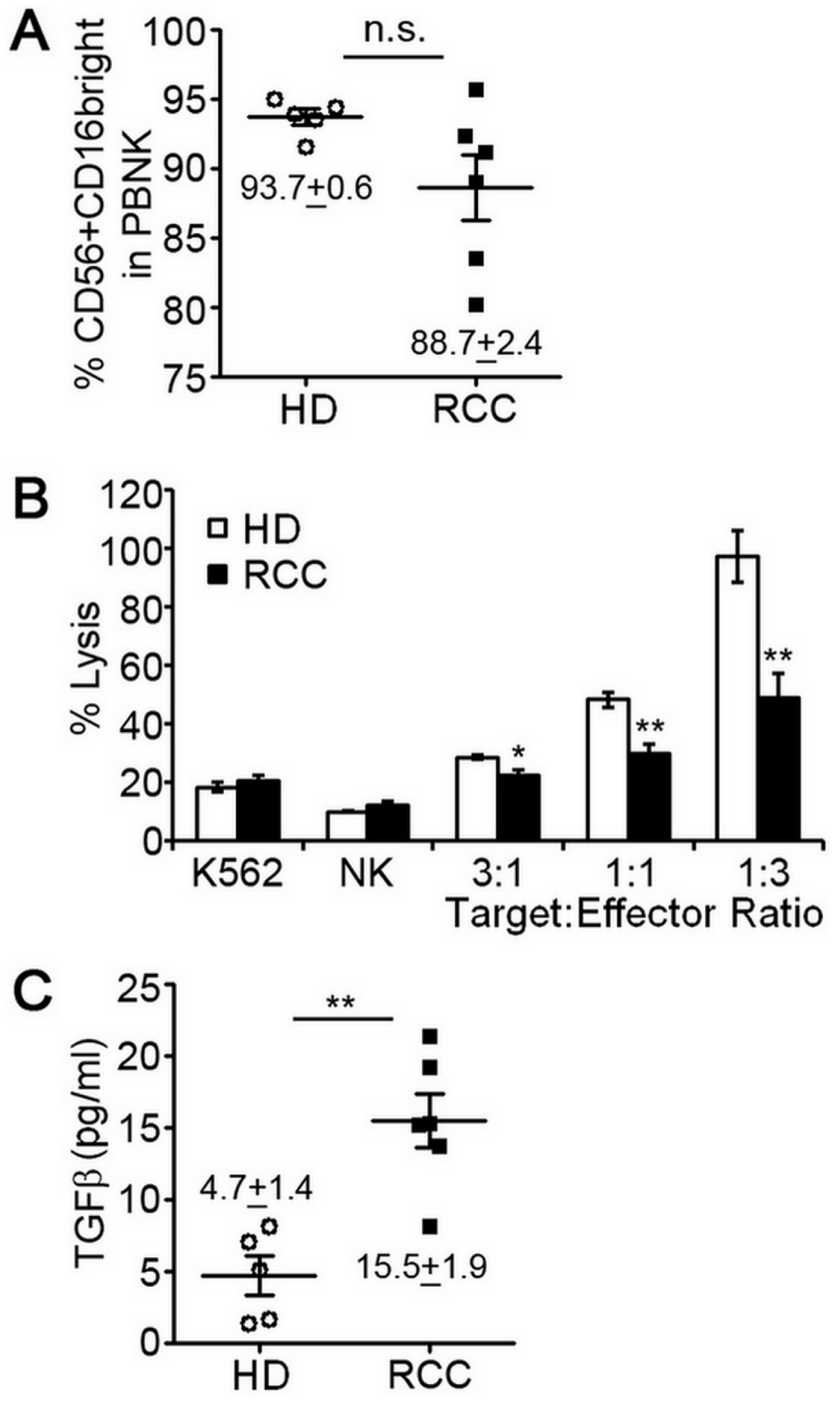
Peripheral blood NK cells of RCC patients have decreased cytotoxic function. NK cells were isolated from peripheral blood of 5 healthy, cancer-free donors (HD) or 6 patients diagnosed with RCC by negative selection and evaluated for expression of surface markers by multi-color flow cytometry or ability to lyse K562 human erythroleukemia cells. (**A**) Percentage of CD56^+^CD16^+^ pNK cells isolated from HD or RCC patients with mean ± SEM reported. Each symbol represents an independent person. (**B**) Relative cytotoxic activity of freshly isolated pNK cells from HD or RCC patients for the indicated target:effector ratios. Data are analyzed relative to K562 cells treated with digitonin serving as a positive control for cell death (set = 100% lysis) and plotted as mean ± SEM (*n* = 5 healthy donors and RCC patients). (**C**) Concentrations of activated TGFβ in plasma from healthy donors and RCC patients determined by ELISA with mean ± SEM reported. Each symbol represents an independent person. n.s., not significant; ^*^ *P* ≤ 0.05; ^**^ *P* ≤ 0.01; determined by Student’s *t*-test.

A role for tumor immunity in controlling RCC has been implicated following rare clinical observations of spontaneous disease regression. The majority of these cases involved regression of metastases after removal of the primary tumor [16, 17]. Puzzling, however, were findings that high levels of tumor infiltrating lymphocytes (TIL), including NK cells, are common to RCC [13–15]. We hypothesized that NK cells of RCC patients were phenotypically and functionally altered within the circulation and/or tumor environment resulting in impaired tumor immunity. To explore the phenotype and function of NK cells in patients with RCC, we enrolled six patients newly diagnosed with this cancer. All patients were males between the age of 50 and 70 years old and none had received prior treatment for the disease. The clinical characteristics of these patients are shown in Table 1. NK cells were isolated from peripheral blood mononuclear cells (PBMC) by negative selection using a cocktail of antibody-magnetic microbeads. Multi-color flow cytometry was used to identify NK cells (CD45^+^/CD3^-^/CD56^+^ cell population) and gauge expression of CD16. Like pNK cells of healthy, cancer-free donors, the CD56^+^CD16^+^ NK subset was predominant for RCC patients (Figure 1A). We did not observe a significant difference in the mean percentage of CD56^+^CD16^+^ NK cells between healthy donors and RCC patients, but there was variation in proportion of CD56^+^CD16^+^ cells among RCC patients. Cytotoxic activity of freshly-isolated pNK cells of RCC patients was, however, significantly reduced compared to healthy controls at all tested target:effector ratios (Figure 1B). As TGFβ treatment has been reported to reduce cytotoxic ability of NK cells [18], we measured the amount of TGFβ in the plasma of RCC patients by ELISA. Compared to healthy volunteers, TGFβ levels were 3-fold higher for RCC patients (Figure 1C). These findings suggest that production of TGFβ by RCC tumors impacts the function of pNK cells in the circulation.

**Table 1:** Characteristics of patients with resected RCC tumors analyzed

No.	Age	Histology	Stage	Tumor Sample (g)	Total Yield (×10^6^)^‡^	CD45+ (%)^#^	CD56+ (%)^^^
**1**	70	Clear Cell	III	1.3	30	74.4	4.1
**2**	55	Clear Cell	III	2.6	10	89.9	36.8
**3**	67	Clear Cell	III–IV	3.6	20	99.0	23.9
**4**	50	Clear Cell	III–IV	1.1	1.7	99.6	1.8
**5**	59	Clear Cell	IV	3.0	20	82.8	34.9
**6**	66	Clear Cell	I–II	0.8	1.7	97.4	6.0

^‡^Total number of cells recovered following centrifugation of dissociated tumor tissue through 35% Percoll gradient. ^#^Percentage of recovered cells that were CD45 positive by flow cytometry. ^^^Percentage of CD45 positive cells that co expressed CD56 when analyzed by flow cytometry.

### Human RCC tumor-derived NK cells acquire altered phenotype and gene expression

**Figure 2:**
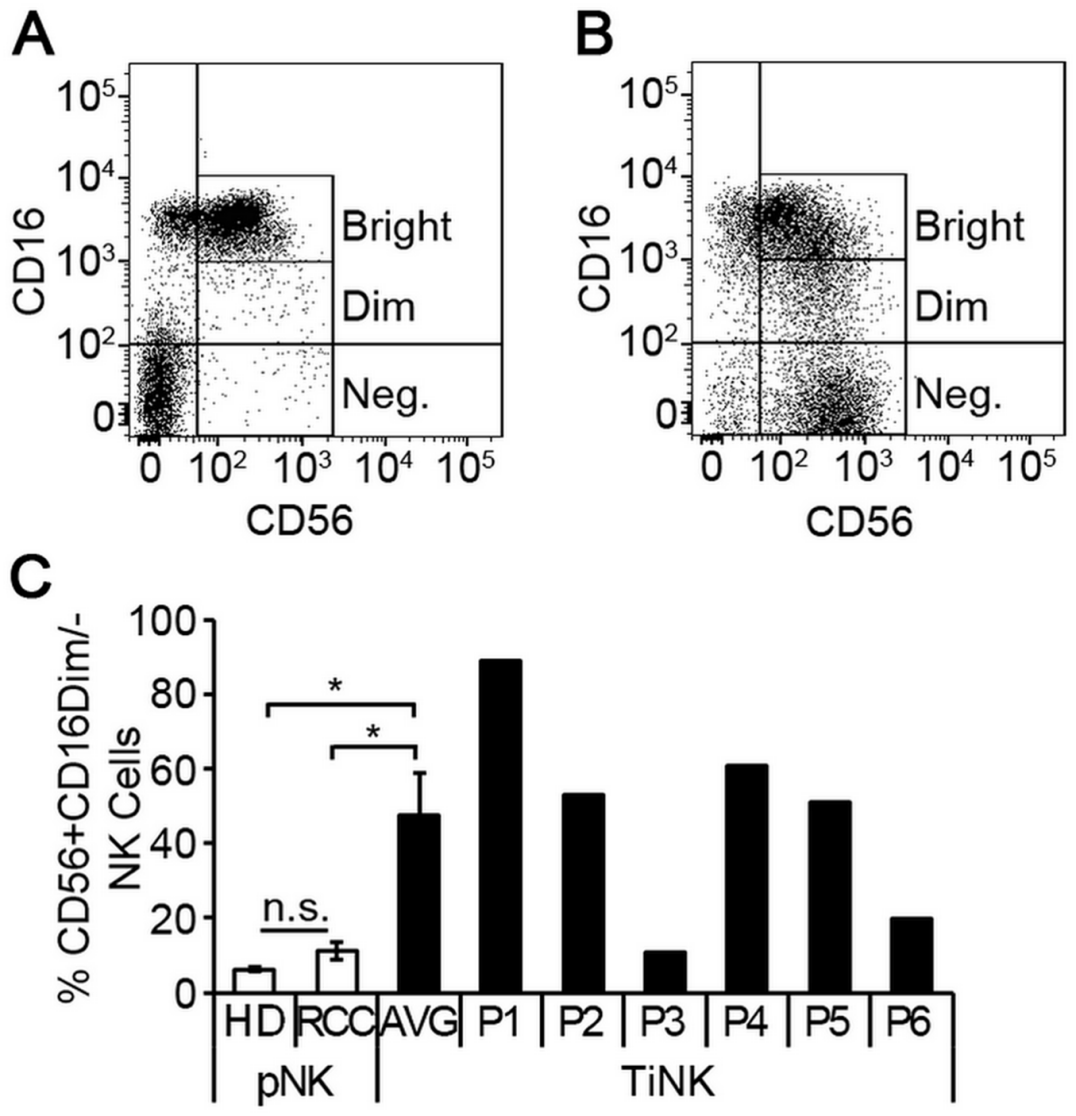
RCC tumor-infiltrated NK cells are phenotypically distinct from matched peripheral blood counterparts. NK cells were isolated from peripheral blood or resected tumor tissues of 6 RCC patients by negative selection and evaluated for expression of CD56 and CD16 by multi-color flow cytometry. Representative dot plots of NK cells isolated from peripheral blood (**A**) versus RCC tumor tissue (**B**) demonstrating CD56 and CD16 expression. (**C**) Percentage of CD56^+^CD16^dim/-^ NK cells for peripheral blood of healthy donors (*n* = 5) and RCC patients (*n* = 6), or RCC tumor-infiltrating NK cells (TiNK, *n* = 6) plotted as mean ± SEM. Results for TiNK cells are also shown for each individual patient (P1 to P6). n.s., not significant; ^*^ *P* ≤ 0.05 determined by Student’s *t*-test.

To identify alterations that were RCC tumor-induced, we compared NK cells isolated from RCC tumors (TiNK) to matched pNK cells for the six patients. The proportion of TiNK cells averaged 18% (range 1.8% to 36.8%) of the total CD45^+^ population (Table 1), which is consistent with previous reports of RCC tumors being infiltrated by lymphocytes, including NK cells [13–15]. Flow cytometry analysis of NK cells isolated from peripheral blood (Figure 2A) or RCC tumor tissue (Figure 2B) revealed that the CD56^+^CD16^dim/neg^ population was significantly enriched for RCC tumors, with mean levels nearly 8-times higher (pNK 6.3% vs. TiNK 47.5%), although variation was noted for individual patients (Figure 2C).

**Figure 3:**
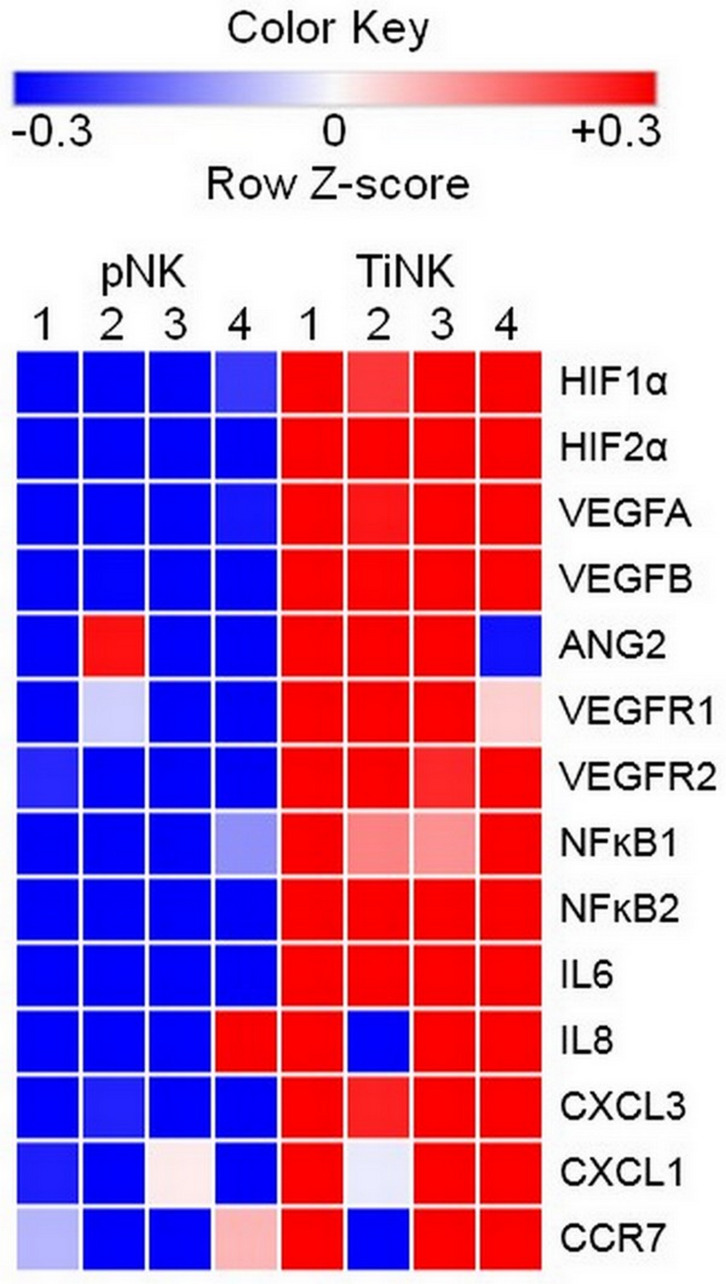
RCC TiNK cells have an altered transcriptional profiled compared to patient matched pNK cells. NK cells isolated from peripheral blood or RCC tumor tissues of 4 patients were isolated of total RNA and RT-qPCR analysis of the indicated targets performed in triplicate. Heat maps of transcriptional changes were developed for calculated Z-scores. Each row corresponds to the listed gene and columns to an individual patient (1–4) with source of NK cells peripheral blood (pNK) or RCC tumor (TiNK) indicated at the top. Scale bar with pseudocolors denotes differential gene expression: blue and red indicate low and high expression, respectively; white indicates no change in expression levels.

To explore molecular signatures that characterized TiNK cells, we used total RNA isolated from purified pNK and TiNK cells of four patients to perform a focused RT-qPCR array consisting of 79 genes with known association to angiogenesis and inflammation. The percentage of the CD56^+^CD16^dim/neg^ population for NK cells isolated from peripheral blood or RCC tumor tissue of these four patients are reported in Supplementary Table 1. RNA samples were analyzed in triplicate and expression level calculated as percentage of β-actin because transcript levels for this gene were essentially unchanged for pNK and TiNK populations (*p* = 0.92 by *t*-test). Supplementary Table 2 shows the results for all 79 genes as mean fold-change ± SEM and ordered from greatest to least fold-change. As defined by (*P* ≤ 0.05 and mean fold-change ≥ 5), 42 out of 79 tested genes were upregulated for TiNK versus pNK populations. Figure 3 shows a heat map depicting differential expression of selected upregulated genes for pNK versus TiNK cells based on calculated Z-scores. KEGG pathway analysis showed that upregulated genes were enriched in pathways related with HIF1, TNF, NFĸB, and transcriptional misregulation in cancer with HIF1 signaling demonstrating the greatest significance (Table 2). In line with this finding, mRNA levels of proangiogenic VEGF were significantly elevated for TiNK versus pNK cell populations from these patients (Supplementary Table 1). Thus, RCC tumor-infiltrating NK cells have pronounced phenotypic and functional alterations compared with matched pNK cells; effects that are likely influenced by the tumor microenvironment.

**Table 2:** Significant signaling pathways based on KEGG database

KEGG ID	Category	Genes in Pathway	*P*-value
**04066**	HIF1 signaling	HIF1α, VEGFA, FLT1, ANG1, ANG2, NFĸB1, RELA, BCL2, IL6	2.1e-8
**04668**	TNF signaling	TNF, NFkB1, IL1β, RELA, CXCL1, CXCL3, CCL20, IL6	7.7e-7
**04064**	NF kappa B signaling	NFĸB1, NFĸB2, TNF, RELA, CCL19, IL1β, BCL2,	2.0e-7
**05202**	Misregulation in cancer	IL3, IL6, CCR7, RELA, IL1β, NFĸB1	1.4e-6

Abbreviations: KEGG, Kyoto encyclopedia of genes and genomes.

### Hypoxia conversion of pNK cells to dNK-like cells

**Figure 4:**
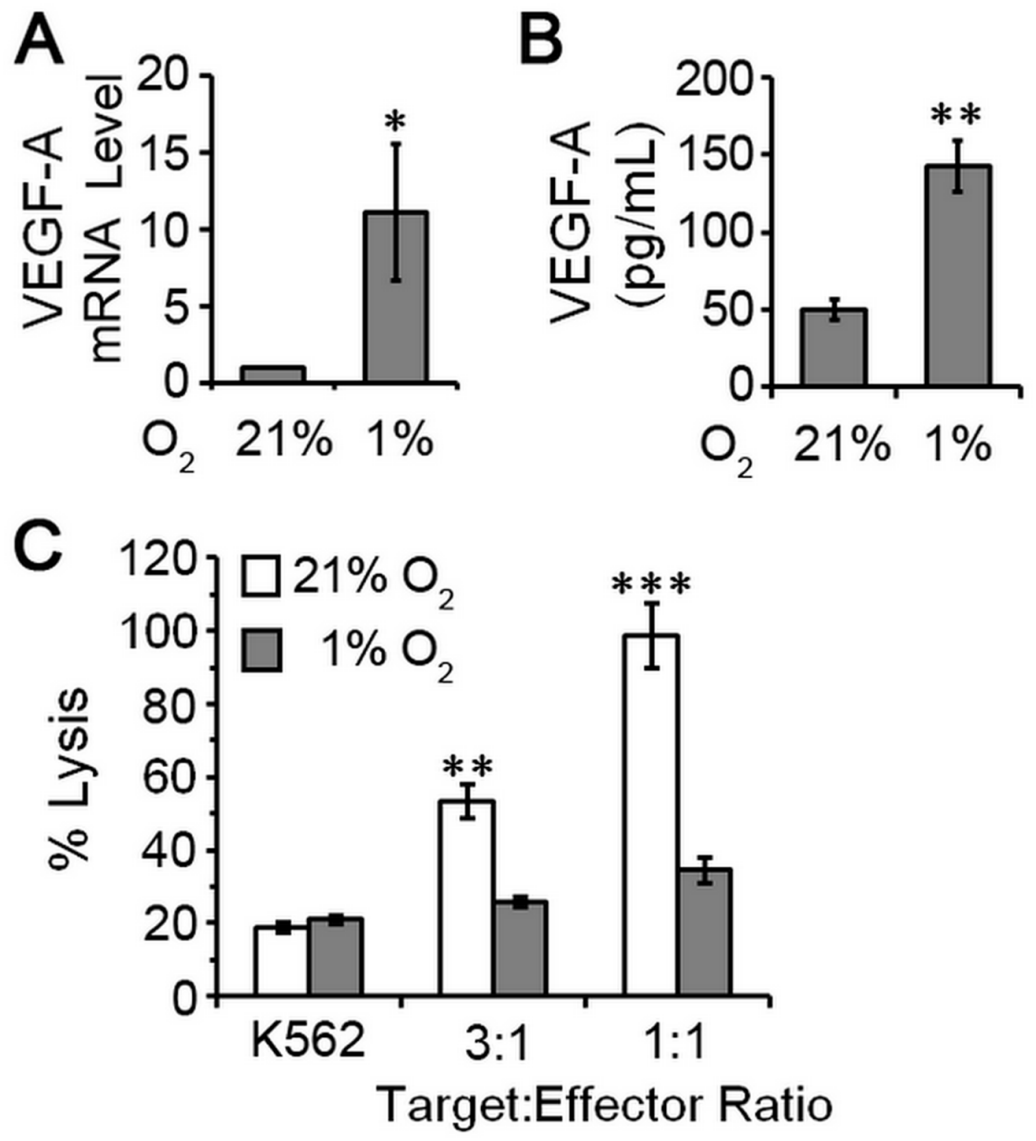
pNK cells exposed to hypoxia are poorly cytotoxic and proangiogenic. NK cells were isolated from peripheral blood of five healthy, cancer-free donors by negative selection and cultured under atmospheres consisting of 21% O_2_ (normoxia) or 1% O_2_ (hypoxia). (**A**) Relative levels of VEGF mRNA for NK cells cultured for 4 days. NK cells maintained under 21% O_2_ conditions set to 1 for normalization and data plotted as mean ± SEM (*n* = 5 donors). (**B**) VEGF secretion by pNK cells cultured under the indicated conditions quantified by ELISA of conditioned supernatants. Data plotted as mean ± SEM (*n* = 5 donors). (**C**) Cytotoxic activity of pNK cells after 4 days of culture under the indicated oxygen conditions. Data are analyzed relative to K562 cells treated with digitonin serving as a positive control for cell death (set = 100% lysis) and plotted as mean ± SEM (*n* = 5 donors). ^*^ *P* ≤ 0.05; ^**^ *P* ≤ 0.01; ^***^ *P* ≤ 0.001 determined by one-way ANOVA with Newman-Keuls post-hoc analysis or paired Student’s *t*-test.

Advanced tumors have abnormal vasculature and insufficient oxygen supply making hypoxia a common feature of the tumor environment [19]. Hypoxia contributes to malignant progression in cancer by inducing an invasive and metastatic phenotype and by activating resistance mechanisms to create an immunosuppressive environment [20]. To test the extent to which hypoxia could enhance the proangiogenic phenotype of pNK cells, we cultured pNK cells (*n* = 5 healthy donors) for four days under normal oxygen (21% O_2_) or hypoxia (1% O_2_), and assayed for proangiogenic VEGFA expression and cytotoxic potential. Because hypoxia can influence cell survival, trypan blue exclusion assay was used to confirm comparable numbers of viable cells under these growth conditions. Thus, changes in gene expression and cytotoxic ability were unrelated to differences cell viability. VEGFA mRNA and protein were expressed at low levels for NK cells cultured under normoxic conditions. Hypoxia clearly affected NK cells as evidenced by well-known upregulation of VEGFA mRNA with mean levels increased 11-fold when quantified by RT-qPCR (Figure 4A). ELISA of conditioned culture supernatants confirmed enhanced production of VEGFA under hypoxic growth conditions (51 pg/mL 21% O_2_ vs. 143 pg/mL 1% O_2_; Figure 4B). Concomitantly, we observed decreased cytotoxicity (Figure 4C), which may result from VEGFA upregulation and/or other HIF1α-regulated factors (Supplementary Figure 1) [21–23]. Thus, conversion of pNK cells to a dNK-like phenotype (poor cytotoxic potential and elaboration of VEGFA expression) is favored by hypoxia; a factor with key roles in tumor invasion and metastasis and response to therapy [19, 20].

### RCC tumor NK cells and decidua NK cells have a shared transcriptional profile

**Figure 5:**
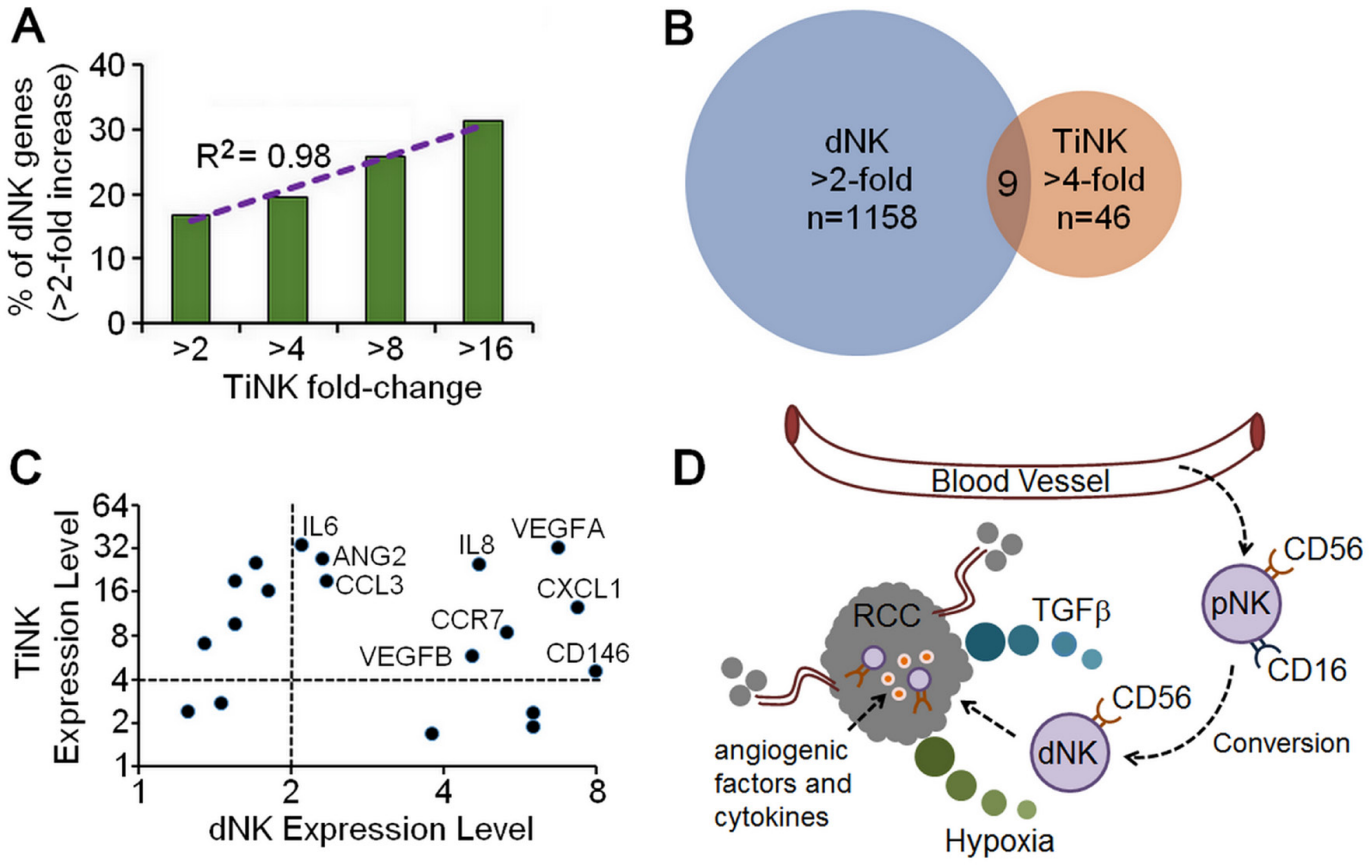
Similarities in gene expression for RCC TiNK and dNK cells. (**A**) Comparison of genes from a published microarray analysis for purified dNK cells [5] with any increase from the minimum hybridization threshold of 20 plotted against genes increased by 2-, 4-, 8-, or 16-fold by RT-qPCR analysis of TiNK cells with regression analysis. (**B**) Venn diagram of gene expression data for dNK cells (microarray data, >2-fold increase) versus RCC TiNK cells (RT-qPCR data, >4-fold increase). (**C**) Genes with any increase above the minimum hybridization threshold of 20 for dNK cells plotted against matched counterparts for TiNK cells with genes demonstrating > 2-fold increase for dNK and >4-fold increase for RCC TiNK labeled. (**D**) Schematic diagram of potential role for CD56^+^CD16^-/dim^ dNK-like cells in RCC tumor development and metastasis. The noncytotoxic dNK-like cells are converted from pNK phenotype in the tumor environment when encountering low oxygen (hypoxia) and/or TGFβ. This results in loss of cytotoxic function and production of angiogenic factors and cytokines supporting tumor growth and metastasis.

Decidua NK cells undergo tissue-specific alterations, which impart their unique ability to regulate vascular remodeling, an essential step for placental growth [24, 25]. Previously, Hanna and colleagues performed a microarray analysis on purified dNK cells to obtain a transcriptional profile of cytokines, chemokines and growth factors using CodeLinkTM Uniset Human 20K I Bioarray, which provides coverage for about 20,000 human genes [5]. To gain additional insight into the function of RCC TiNK cells, we compared gene expression results for our 79 genes to these microarray data (Supplementary Table 3). We first looked at genes with any increase above the minimum hybridization threshold of 20 for dNK cells versus genes increased by 2-, 4-, 8-, or 16-fold for TiNK cells. This analysis revealed that as the level of gene expression increased for TiNK cells it became more likely that these same genes were upregulated in dNK cells (R^2^ = 0.98) (Figure 5A). Comparison of genes induced by at least 2-fold for dNK cells and at least 4-fold for TiNK cells identified 1,158 genes for dNK cells and 46 for TiNK cells with 9 genes present in both data sets (Figure 5B). Genes with any increase above the minimum threshold of 20 for dNK cells were also plotted against matched counterparts for TiNK cells (Figure 5C). This comparison identified genes that were relatively non-specific, specific to dNK or TiNK, or shared by both dNK and TiNK. It is important to note that CD146 was significantly increased for dNK cells (53.8-fold increase over the minimum threshold), but assigned a value of 8 for visualization purposes in the graphical representation. For the 9 shared genes, the majority have known roles in angiogenesis, immune modulation, tumor development, and/or metastasis (Table 3). As angiogenesis and immune modulation are critical features of tumor and decidua environments, these gene expression results provide further evidence for similarities between dNK and RCC TiNK cells.

**Table 3:** Function of genes upregulated in dNK and TiNK cells

Gene	Description	Function	Ref.
VEGFA	Growth Factor	Physiological/pathological angiogenesis; vascular permeability; tumorigenesis	[62]
VEGFB	Growth Factor	Physiological/pathological angiogenesis; maintenance of blood vessels	[63]
ANG2	Growth Factor	Physiological/pathological angiogenesis; vascular permeability; tumorigenesis	[64]
IL6	Cytokine	Acute and chronic inflammation; leukocyte recruitment	[65]
IL8	Cytokine	Acute and chronic inflammation; neutrophil recruitment	[66]
CCL3	Chemokine	Acute inflammation; leukocyte recruitment	[67]
CXCL1	Chemokine	Angiogenesis, inflammation, wound healing	[68]
CCR7	Receptor	Homing to lymphoid organs; recognizes CCL19 and CCL21 ligands	[69]
CD146	Receptor	NK cell adhesion; angiogenesis; immune response	[70]

## DISCUSSION

A subset of non-classical NK cells (CD56^+^CD16^neg^; dNK cells) has become recognized for its role in vascular remodeling and tissue construction. Most knowledge of this angiogenic potential comes from reproductive biology, where these cells are undeniably important [5–8]. Relevance to cancer is evidenced by the ability of purified dNK cells to enhance growth of JEG3 choriocarcinoma cells when co-inoculated into immune deficient nude mice [5]. Examples of NK cells with a similar phenotype exist for cancer patients, including increased levels of CD56^+^CD16^dim/neg^ cells in tumor specimens from patients with breast cancer [2], non-small cell lung cancer (NSCLC) [3, 26], and colorectal cancer [27, 28]. Increased percentages of CD56^+^CD16^dim/neg^ NK cells in the peripheral blood of some patients with these cancers suggest phenotype-altering effects can extend beyond the local tumor environment and may be indicative of disease progression and metastasis [2, 26–28]. Here we show that NK cells were altered within human RCC tumors compared with autologous peripheral blood samples. NK cells in the blood of RCC patients were poorly cytotoxic even though phenotypic alterations were less pronounced. Although not statistically significant in our small sample size, these changes could reflect what occurs in the tumor. These data support a model in which pNK with the decidua-like phenotype are either directly recruited to RCC tumor sites or converted to phenotypically and functionally resemble dNK cells in the hypoxic tumor environment when encountering TGFβ to promote tumor establishment, growth and/or metastasis (Figure 5D).

TGFβ is highly expressed in the decidua *in situ* [29] and several studies have reported exposure of pNK cells from healthy donors to TGFβ mediated conversion to dNK-like cells [30, 31]. TGFβ is also detected at high levels in various tumors and is the major immunosuppressive cytokine in the tumor microenvironment [32]. In RCC, TGFβ expression directly correlates with tumor stage and grade and is significantly elevated with metastatic disease [33, 34], suggesting its importance in tumor progression, immune evasion and potential role in transformation of TiNK cells [4, 35]. Plasma TGFβ levels are upregulated in lung [36] and colon cancer patients [37]. The RCC patients studied here had 3-fold higher levels of TGFβ in the blood compared with healthy cancer-free volunteers. Circulating NK cells of RCC patients were phenotypical similar to healthy donors, but lacked full cytotoxic ability which we hypothesize is attributed to heightened levels of TGFβ. A more pronounced diminution of cytotoxicity was observed for RCC TiNK cells which were predominantly dNK-like (CD56^+^CD16^dim/neg^). TGFβ has been associated with NK cell dysfunction and changes in expression of activating and inhibitory receptors have been described in patients with breast cancer, melanoma, lung cancer and ovarian carcinoma [2–4, 35–38]. Many types of cells at the tumor site, including tumor cells, fibroblasts, stromal cells and infiltrating immune cells, express TGFβ to promote an immunosuppressive tumor microenvironment [32, 35]. A recent report demonstrated that myeloid-derived suppressor cells (MDSC) from patients with advanced melanoma suppress NK cell activity through the production of TGFβ [39]. This would be in keeping with the immunomodulatory role of TGFβ in several tumors and tumor models [28, 32, 40], similar to what occurs for dNK cells [5, 29, 41]. Thus, TGFβ, in combination with other factors and/or stimuli, such as hypoxia, may result in conversion of NK cells to an angiogenic phenotype within the decidua and tumor microenvironment. In support of this notion, inhibition of TGFβ has been reported to preserve function of activated and *ex vivo* expanded NK cells in tumor models [42].

A distinct feature of RCC is high numbers of tumor infiltrating lymphocytes. Consistent with previous reports [13–15], NK cells were present in the RCC tumors studied here, but percentages varied among patients and did not seem to correlate with pathologist-assigned staging criteria. RCC TiNK cells displayed the dNK-like phenotype and a distinct gene expression profile compared to peripheral blood counterparts. Among the upregulated genes, a number of genes within the HIF1 pathway were highly enriched, including the proangiogenic molecules, VEGFA, VEGFB, and ANG2. Thus, the NK cells isolated from the RCC tumors represented truly infiltrating populations and not peripheral blood contaminants through surrounding vasculature. Consistent with previous reports [22, 23], we demonstrated that hypoxia decreases NK cell cytotoxicity. Hypoxia also resulted in dramatic up-regulation of VEGF. Increased expression of VEGF in tumors and pathological angiogenesis is correlated with poor prognosis [43–45]. These findings confirm the association between phenotypic markers (CD56^+^CD16^neg^) and the ability to produce proangiogenic factors.

The presence of the RCC tumor appears to have a systemic effect on the cytotoxic activity of the pNK cell by augmenting circulating levels of TGFβ. The observation that CD56^+^CD16^+^ NK cells remain the dominate population in the peripheral blood of RCC patients suggests counter mechanisms are in play to retain surface markers, but these effects are less capable of preserving full cytotoxic function. Our observations for RCC TiNK are consistent with findings that a CD56^+^CD16^neg^ subset of NK dominates in NSCLC tumors acting as proangiogenic cells by producing VEGF and PGF [26]. While other factors may contribute to the observed conversion of RCC TiNK cells, we have shown that TGFβ and hypoxia are two likely candidates. In support of this assertion, blocking TGFβ signaling can overcome immune suppression and enhance the effects of NK cell therapy [46, 47]. As TGFβ is a pleotropic cytokine with important biological function, it is also intriguing to assume that proangiogenic function of RCC TiNK cells could be suppressed by targeted VEGF blocking agents including antibodies (i. e., bevacizumab) and tyrosine kinase inhibitors (TKIs), such as sunitinib and sorafenib. These medicines are already used clinically and could ameliorate or reverse tumor-supportive function of TiNK cells.

NK cell-mediated antitumor activity is commonly described and suggests these cells could be useful in therapeutic approaches. For RCC, rare patients with metastatic disease can experience spontaneous remission supporting the notion that RCC could be immunologically controlled [16, 17]. IL2, IL15, IL12, and IFNα (alpha interferon) have demonstrated some efficacy, with improved success noted when used in combination, however, response rates have been limited (ranging from 15–20%) [11, 48–51]. The precise mechanisms by which these cytokines exert their antitumor effects are unknown, but it is believed activation and expansion of T cells and NK cells has a pivotal role [11, 12, 28, 42]. Of particular relevance to our studies are findings that IL12 has the ability to rescue NK cell antitumor activity by upregulation of CD16 [52]. As an alternative to cytokine stimulation of endogenous NK cells, other efforts have explored the benefit of NK cell infusion for RCC. Early studies tested tumor regressive properties of lymphokine activated CD3^−^CD56^+^ cells (or LAKs) injected in combination with IL-2 [53, 54]. Follow-up studies employed adoptive transfer of *ex vivo* activated allogeneic NK cells [55] and NK cell lines, such as NK-92 [56, 57]. NK cells have also been genetically modified to reduce expression of inhibitory receptors or augment production of cytokines or activating receptors. For example, NK cells engineered to express CXCR2 demonstrated improved trafficking and cytotoxic potential [58]. Chimeric antigen receptor (CAR) engineered NK-92 cells expressing a CAR specific to ErbB2 (Her2) or EGFR have shown efficacy in mouse models of human RCC [59, 60], which suggest opportunities for future benefit of CAR-NK cells in patients [12, 35, 56].

Tumor-induced alterations of NK cells can limit tumor cell recognition and decrease their ability to interact with other immune cells. We showed that TGFβ and hypoxia are (at least) two factors capable of supporting the conversion of pNK cells to a dNK-like phenotype within RCC tumors. While these characteristics are conceivably beneficial for placentation, they may be exploited to support RCC growth and metastasis. Our findings that peripheral blood of RCC patients has higher levels of TGFβ and less cytotoxic NK cells suggests these parameters could be used to monitor disease progression. The utility of these criteria will require assessment of patients with early (stage I/II) versus advanced (stage III/IV) disease, which is challenging in RCC as the majority of patients are in advanced stage at time of presentation. The distinct gene expression signature of RCC TiNK cells discovered here provides additional targets responsible for proangiogenic differentiation of NK cells in the tumor environment. Improved knowledge of the extent and mechanisms of these newly-identified targets could permit development of strategies to restore the ability of NK cells to recognize and lyse tumors, particularly in patients with advanced disease. Along these lines, it is interesting to consider that combined use of TGFβ inhibitors and targeted VEGF blocking agents could ameliorate or reverse tumor-supportive function of TiNK cells. These and related studies in RCC and other cancer types may provide foundations for far reaching benefits in diseases in which inhibition or augmentation of vascular growth is warranted.

## MATERIALS AND METHODS

### Human samples and processing

Specimens from healthy donors (venous blood) and patients diagnosed with RCC (venous blood and tumor tissue) were prospectively collected with the donor’s written informed consent in accordance with protocols approved by the Springfield Committee for Research Involving Human Subjects (SCRIHS) of Southern Illinois University School of Medicine. For RCC patients, morphological tumor characteristics were evaluated by a pathologist before release to the laboratory. All specimens lacked personal health information (de-identified) and were processed on the collection day.

Blood (10–20 mL) was diluted 1:2 with phosphate buffered saline (PBS), layered over Ficoll-Paque Plus (1.077 density, GE Healthcare, Uppsala, Sweden) and mononuclear cells separated by centrifugation at 300 rcf for 30 minutes. RCC tumor tissues were minced and disrupted with a gentle MACS dissociator (program hTumor-01; Miltenyi Biotec, San Diego, CA, USA). Tissue fragments were incubated with RPMI 1640 medium supplemented with collagenase (1 mg/mL; Sigma-Aldrich, St. Louis, MO, USA), DNase I (100 μg/mL; Roche NimbleGen Inc., Madison, WI, USA), 50 units/ml each penicillin and streptomycin for 1 hour at 37°C. The digested mixture was filtered through a 70-μm nylon cell strainer (BD Falcon, Franklin Lakes, NJ, USA) and cells collected by centrifugation at 500xg. Pelleted cells were suspended in 35% Percoll solution (Sigma Aldrich, St. Louis, MO, USA) and centrifuged at 300 rcf for 30 minutes. Cells recovered from Ficoll or Percoll were washed twice with phosphate buffered saline (PBS). Viable cell counts were performed by trypan blue exclusion assay and cells were prepared for flow cytometry analysis or NK isolation as detailed below.

### NK cell isolation

NK cells were isolated by negative selection (Dynabeads Untouched hNK Cell kit, Thermo Fisher Scientific, Grand Island, NY, USA) according to the manufacturer’s instructions. The antibody-magnetic microbead cocktail removes T cells, B cells, NKT cells, dendritic cells, platelets, monocytes, granulocytes and erythroid cells to yield a population of NK cells that are bead- and antibody-free. The viability and purity of isolated NK cells was greater than 90% as determined by trypan blue exclusion assay and flow cytometry, respectively.

### NK cell phenotype by flow cytometry

Purified NK cells were reacted with APC-conjugated anti-CD45, PE-conjugated anti-CD3, Alexa 488-conjugated anti-CD56, and APCH7-conjugated anti-CD16 in PBS supplemented with 0.5% BSA for 30 min on ice and washed three times. Viable cells were selected by gating on propidium iodide negative populations. NK cells were identified as the CD45^+^/CD3^-^/CD56^+^ population and evaluated for expression of CD16. Cells left unstained or reacted with isotype control antibodies served as negative controls for gating. All antibodies were purchased from BD Biosciences (San Jose, CA, USA). Flow cytometry was performed on FACSAriaII (BD Biosciences) and analysis completed using FlowJo v10.0 software (FLOWJO, LLC, Ashland, OR, USA). Data are expressed as logarithmic values of fluorescence intensity.

### NK cell culture

NK cells were cultured at 10^6^ cells/mL in Myelocult medium (Stem Cell Technologies, Vancouver, BC, Canada) containing 10% human serum, 5% fetal calf serum, 20 ng/mL human interleukin-15 (hIL-15; PeproTech, Rocky Hill, NJ, USA), 20 ng/mL human stem cell factor (hSCF; PeproTech) and 10^−6^ M hydrocortisone (Stem Cell Technologies, Vancouver, BC, Canada). Cells were cultured for four days under 21% or 1% O_2_ in a humidified atmosphere at 37°C.

### NK cell cytotoxic activity assays

Human K562 erythroleukemia cells (CCL-243, ATCC, Manassas, VA, USA) were used to assay for NK activity. K562 cells were cultured in Iscove’s Modified Dulbecco’s Medium (IMDM; Mediatech Inc., Manassas, VA, USA) supplemented with 50 units/ml each penicillin and streptomycin, and 10% heat-inactivated fetal bovine serum (FBS) all from Hyclone Laboratories (Logan, UT, USA) at 37°C in a humidified 5% CO_2_ atmosphere. Freshly isolated pNK cells (effectors) of healthy donors or RCC patients were evaluated for cytotoxic capacity using K562 (targets) and the Multi-Tox Fluor Cytoxicity Assay (Promega, Madison, WI, USA). K562 and NK cells were plated in triplicate into 96-well round bottom culture plates (BD Falcon, Franklin Lakes, NJ, USA) to achieve target:effector (T:E) ratios of 3:1, 1:1, and 1:3, respectively, in a total volume of 100 μL of IMDM. Control wells containing only K562 or NK cells were included to measure spontaneous cell death whereas culture medium was assayed to control for background absorbance. K562 cells treated with the non-ionic detergent, Digitonin (Promega), at a final concentration of 30 μg/mL were included as positive controls for cell death. After 4 hours of incubation at 37°C in a humidified 5% CO_2_ atmosphere, cells were reacted with bis-AAF-R110 substrate which is cleaved by the dead cell protease to release R110. The free R110 results in the formation of a yellow product that is quantitated by measuring fluorescence at 520 nm on a Glomax multimode plate reader (Promega). The amount of fluorescence is proportional to the number of lysed cells and percent cytotoxicity calculated using the equation: % cytotoxicity = [(Experimental – Effector Spontaneous – Target Spontaneous)/(Target Maximum – Target Spontaneous)] × 100.

### Quantitative RT-PCR

NK cells isolated from RCC patient blood or tumor tissue were extracted of total RNA using Ambion spin columns (Life Technologies, Carlsbad, CA, USA) with on-column DNase treatment (Promega). RNA was quantified by a Nanodrop 2000 (Life Technologies) and quality assessed by visualizing 18S and 28S ribosomal RNA bands separated through 1% agarose. RNA (100–300 ng) was reverse transcribed into cDNA using SuperScript VILO cDNA synthesis kit (Thermo Fisher Scientific, Waltham, MA, USA) and conditions: 25°C-10 min, 42°C-60 min, 85°C-5 min, and 4°C-hold.

Differential gene expression was determined using cDNA (100 ng/reaction) as template and primer pairs for 79 genes selected from an in-house gene array. The details of this array including primer sequences and amplicon size have been described in detail [61]. For this study, interrogated genes included cytokines/chemokines/growth factors and their receptors; inflammation/activation-associated genes; single transduction/transcription factors; and cell lineage genes (Supplementary Table 2). Quantitative PCR reactions were performed in triplicate with iTaq Universal SYBR Green Supermix (Bio-Rad, Hercules, CA, USA) on a StepOne Plus thermocycler (Thermo Fisher Scientific-Applied Biosystems) using SYBR Green settings that included a final melt curve analysis; all reactions yielded a single peak. Changes in transcript levels were assessed by the ΔΔCt method and data normalized to β-actin. Paired *t*-test was used to identify genes with a fold change ≥ 5.0 and a *P*-value ≤ 0.05, and these genes were selected for the following analyses. Z-scores were calculated for each target gene using the equation Z = [(target – mean)/standard deviation] and a heat map created using Morpheus software (Broad Institute, Cambridge, MA; https://software.broadinstitute.org/morpheus/). The Kyoto encyclopedia of gene and genomes (KEGG) pathway analysis was performed to predict the potential significant signaling pathways involved in conversion of pNK to TiNK (*p* ≤ 0.05).

### ELISA

#### TGFβ

TGFβ content in human serum was evaluated using a TGFβ ELISA Duoset (R&D Systems) following the manufacturer’s instructions. Briefly, to activate latent TGFβ, plasma was incubated with 1 N hydrochloric acid (HCl) followed by neutralization with 1.2 N sodium hydroxide (NaOH)/0.5 M 4-(2-hydroxyethyl)-1-piperazineethanesulfonic acid (HEPES) buffer.

#### VEGF

NK cells were cultured under the indicated conditions for 4 days, and culture supernatants collected and stored at –80°C. VEGF concentration was measured using a VEGF Quantikine ELISA kit (R&D Systems, Minneapolis, MN, USA) according to instructions. Results were normalized to viable cell counts determined at the time of collection.

### Statistical analysis

Microsoft Excel or Prism 5 (GraphPad Software, Inc., La Jolla, CA, USA) was used to determine descriptive statistics (mean ± SD or SEM) and calculate Z-scores. Significant differences between mean values were determined by paired or unpaired Student’s *t*-test (two-tailed) for independent groups or one-way ANOVA with Newman-Keuls post-hoc test for multiple comparisons where indicated. *P*-values are indicated by asterisks in the figures with level of significance reported as ^*^ *P* ≤ 0.05; ^**^ *P* ≤ 0.01; ^***^ *P* ≤ 0.001.

## SUPPLEMENTARY MATERIALS







## Abbreviations

ANG: angiopoietin
CCL: C-C motif chemokine ligand
CCR: C-C motif chemokine receptor
CD: cluster of differentiation
CXCL: C-X-C motif chemokine ligand
CXCR: C-X-C motif chemokine receptor
dNK: decidua natural killer cells
FLT: fems-like tyrosine kinase
GM-CSF: granulocyte macrophage-colony stimulating factor
HIF: hypoxia inducible factor
IL: interleukin
INFα: alpha interferon
IFNγ: gamma interferon
MDSC: myeloid-derived suppressor cells
PBMC: peripheral blood mononuclear cells
PGF: placenta growth factor
pNK: peripheral blood natural killer cells
NK: natural killer
NFĸB: nuclear factor kappa B
RCC: renal cell carcinoma
TGFβ: transforming growth factor beta
TIL: tumor infiltrating lymphocytes
TiNK: tumor-infiltrated natural killer cells
TNFα: tumor necrosis factor alpha
VEGF: vascular endothelial growth factor
