# Supplementary material for: Renal cell tumors convert natural killer cells to a proangiogenic phenotype
Source: Oncotarget. 2020 Jun 30;11(26):2571–85. doi: 10.18632/oncotarget.27654 (PMC7335666; doi:10.18632/oncotarget.27654)
Supplement: Supplementary file 1 [file oncotarget-11-2571-s001.pdf]

Renal cell tumors convert natural killer cells to a proangiogenic phenotype

SUPPLEMENTARY MATERIALS

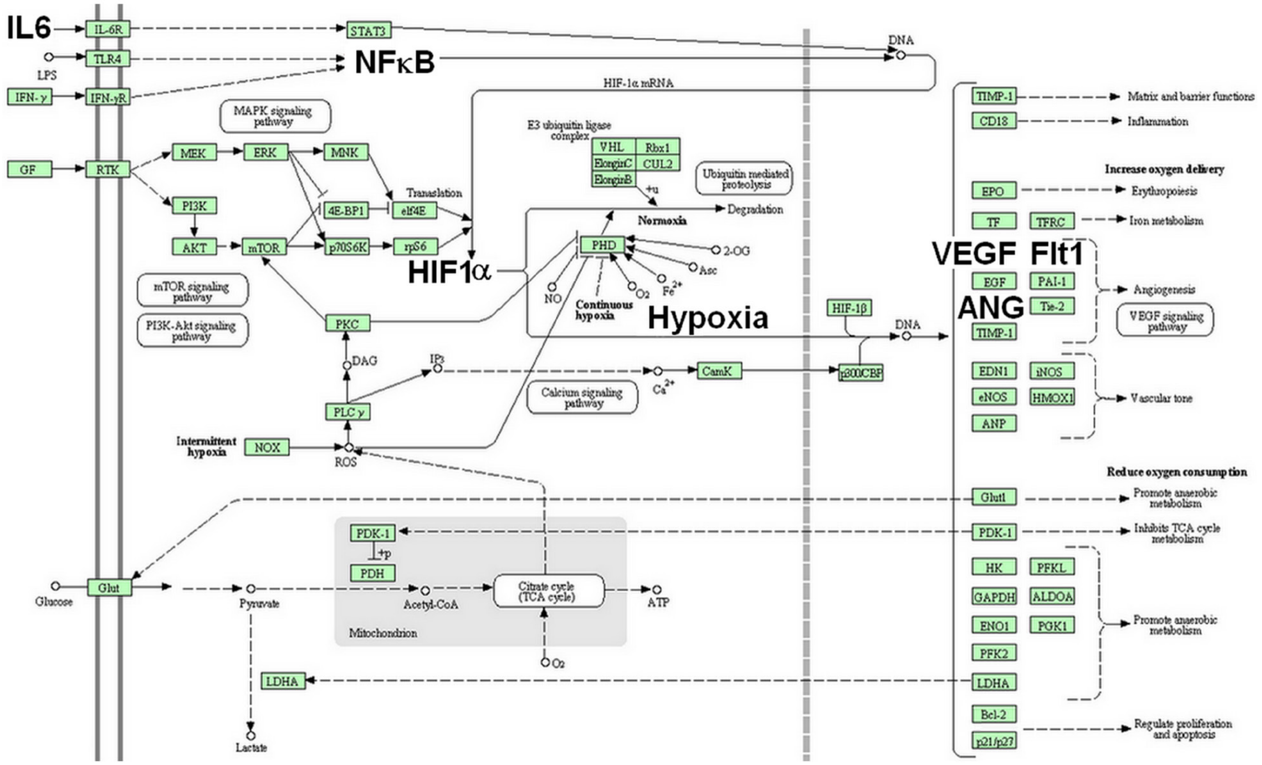

Supplementary Figure 1: HIF1α signaling pathway. Schematic diagram of the HIF1α signaling pathway with genes and characteristics of direct significance to conversion of pNK to dNK-like cells in the tumor microenvironment highlighted in bold.

Supplementary Table 1: Phenotype of patient-matched pNK and TiNK cells used for gene expression studies

	Peripheral Blood (pNK)		RCC Tumor (TiNK)	
	$CD56^{+}CD16^{dim/neg\#}$ (%)	VEGF mRNA† (% β-actin)	$CD56^{+}CD16^{dim/neg\#}$ (%)	VEGF mRNA† (% β-actin)
Pt. 1	20	0.294	89	8.379
Pt. 2	11	0.035	51	3.359
Pt. 3	4	0.076	19	0.645
Pt. 4	8	0.039	61	2.017

#Determined by multi-color flow cytometry and reported as percentage of total $CD56^{+}$ population.
†Determined by RT-qPCR and reported as percentage of β-actin internal control.

Supplementary Table 2: Transcript levels in NK cells isolated from peripheral blood (pNK) or tumor tissue (TiNK) of patients with renal cancer. See Supplementary Table 2

Supplementary Table 3: Gene expression results for RCC NK cells versus dNK cells. See Supplementary Table 3
