# Supplementary material for: Renal cell tumors convert natural killer cells to a proangiogenic phenotype
Source: Oncotarget. 2020 Jun 30;11(26):2571–85. doi: 10.18632/oncotarget.27654 (PMC7335666; doi:10.18632/oncotarget.27654)
Supplement: Supplementary file 2 [file oncotarget-11-2571-s002.docx]

**Supplementary Table 2: Transcript levels in NK cells isolated from peripheral blood (pNK) or tumor tissue (TiNK) of patients with renal cancer**

| Gene | Accession No. | RCC pNK  (% β-actin) | RCC TiNK  (% β-actin) | Fold-Increase | *P*-value |
| --- | --- | --- | --- | --- | --- |
| HIF1α | NM­_001530 | 0.1660 ± 0.0469 | 8.7794 ± 2.9354 | 52.88 | 0.0002 |
| CCL1 | NM_002981 | 0.0216 ± 0.0077 | 0.8968 ± 0.4285 | 41.48 | 0.0023 |
| ANG1 | NM_001146 | 0.0080 ± 0.0047 | 0.2912 ± 0.1322 | 36.44 | 0.0016 |
| IL6 | NM_000600 | 0.0107 ± 0.0025 | 0.3577 ± 0.1088 | 33.57 | 0.0001 |
| VEGFA | NM_001025366 | 0.1108 ± 0.0616 | 3.6000 ± 1.6866 | 32.49 | 0.0019 |
| HIF2α | NM_001430 | 0.0709 ± 0.0051 | 2.2735 ± 0.6780 | 32.05 | 0.0001 |
| IL7 | NM_000880 | 0.0482 ± 0.0187 | 1.4989 ± 0.8324 | 31.12 | 0.0059 |
| ANG2 | NM_001147 | 0.0774 ± 0.0300 | 2.1075 ± 1.1196 | 27.22 | 0.0057 |
| CXCL3 | NM_002090 | 0.0828 ± 0.0318 | 2.1222 ± 1.5542 | 25.63 | 0.0284 |
| IL8 | NM_000584 | 0.1673 ± 0.0490 | 4.1773 ± 1.5567 | 24.96 | 0.0004 |
| NFĸB2 | NM_002502 | 0.0922 ± 0.0642 | 2.2475 ± 1.0361 | 24.36 | 0.0017 |
| CCL19 | NM_006274 | 0.0838 ± 0.0294 | 1.9555 ± 1.0068 | 23.34 | 0.0041 |
| VEGFR1 | NM_002019 | 0.0870 ± 0.0260 | 1.9562 ± 0.8715 | 22.48 | 0.0022 |
| CCL20 | NM_004591 | 0.0298 ± 0.0137 | 0.5966 ± 0.3625 | 20.03 | 0.0114 |
| CCL3 | NM_002983 | 0.0083 ± 0.0018 | 0.1593 ± 0.0732 | 19.14 | 0.0022 |
| TAL1 | NM_003189 | 0.1242 ± 0.0503 | 2.3565 ± 1.2932 | 18.98 | 0.0060 |
| HOXA4 | NM_002141 | 0.1923 ± 0.0753 | 3.1505 ± 1.5549 | 16.38 | 0.0037 |
| TNFα | NM_000594 | 0.0221 ± 0.0073 | 0.3151 ± 0.1266 | 14.29 | 0.0010 |
| HOXA9 | NM_152739 | 0.1090 ± 0.0382 | 1.5564 ± 0.7411 | 14.28 | 0.0027 |
| IL15 | NM_000585 | 0.0117 ± 0.0092 | 0.1637 ± 0.1420 | 14.00 | 0.0510 |
| IL5 | NM_000879 | 0.0563 ± 0.0126 | 0.7603 ± 0.2738 | 13.50 | 0.0003 |
| DLL4 | NM_019074 | 0.0024 ± 0.0003 | 0.0321 ± 0.0146 | 13.42 | 0.0025 |
| NFĸB1 | NM_003998 | 0.1735 ± 0.1416 | 2.2153 ± 0.9695 | 12.77 | 0.0009 |
| IL1β | NM_000576 | 0.0188 ± 0.0061 | 0.2371 ± 0.0842 | 12.61 | 0.0003 |
| CXCL1 | NM_001511 | 0.0586 ± 0.0116 | 0.7290 ± 0.2855 | 12.43 | 0.0008 |
| CD133 | NM_006017 | 0.0047 ± 0.0018 | 0.0555 ± 0.0225 | 11.85 | 0.0013 |
| SIX1 | NM_005982 | 0.0615 ± 0.0356 | 0.6290 ± 0.3596 | 10.22 | 0.0079 |
| RELA | NM_ 021975 | 0.0163 ± 0.0043 | 0.1587 ± 0.0463 | 9.72 | 0.0001 |
| PECAM1 | NM_000442 | 0.0586 ± 0.0077 | 0.5631 ± 0.1681 | 9.61 | 0.0001 |
| CXCR1 | NM_005283 | 0.1570 ± 0.1024 | 1.4546 ± 0.7952 | 9.27 | 0.0060 |
| CD34 | NM_001773 | 0.1929 ± 0.0720 | 1.6451 ± 1.6451 | 8.53 | 0.0039 |
| CCR7 | NM_001838 | 0.3483 ± 0.1603 | 2.9437 ± 2.1046 | 8.45 | 0.0329 |
| IL10R | NM_000628 | 0.0207 ± 0.0093 | 0.1720 ± 0.0895 | 8.32 | 0.0055 |
| CXC3R1 | NM_ 001171174 | 0.2793 ± 0.1402 | 2.1685 ± 1.2425 | 7.76 | 0.0080 |
| IL4 | NM_000589 | 0.0401 ± 0.0207 | 0.2809 ± 0.2302 | 7.01 | 0.0545 |
| CCR1 | NM_001295 | 0.4108 ± 0.1223 | 2.8239 ± 1.3955 | 6.87 | 0.0063 |
| VEGFR2 | NM_002253 | 0.3516 ± 0.0761 | 2.2583 ± 0.7858 | 6.42 | 0.0004 |
| BCL2 | NM_000633 | 0.0454 ± 0.0143 | 0.2842 ± 0.1767 | 6.26 | 0.0229 |
| NFATc1 | NM_006162 | 0.1118 ± 0.0402 | 0.6969 ± 0.2532 | 6.23 | 0.0010 |
| CCR3 | NM_001164680 | 0.6665 ± 0.2454 | 3.9615 ± 1.8161 | 5.94 | 0.0024 |
| VEGFB | NM_003377 | 0.0472 ± 0.0117 | 0.2738 ± 0.0456 | 5.80 | 0.0000 |
| PAX6 | NM_001604 | 0.0861 ± 0.0338 | 0.4903 ± 0.2323 | 5.70 | 0.0063 |
| IL3 | NM_000588 | 0.0474 ± 0.0186 | 0.2423 ± 0.0847 | 5.11 | 0.0005 |
| PAX4 | NM_006193 | 0.0012 ± 0.0004 | 0.0060 ± 0.0013 | 5.02 | 0.00001 |
| IL33 | NM_033439 | 0.0506 ± 0.0133 | 0.2423 ± 0.1271 | 4.79 | 0.0110 |
| IL7R | NM_002185 | 0.4150 ± 0.1669 | 1.9554 ± 1.2149 | 4.71 | 0.0186 |
| CD146 | NM_006500 | 0.0054 ± 0.0019 | 0.0245 ± 0.0131 | 4.52 | 0.0079 |
| IL3R | NM_002183 | 0.0142 ± 0.0075 | 0.0610 ± 0.0300 | 4.30 | 0.0030 |
| TEAD2 | NM_003598 | 0.0018 ± 0.0010 | 0.0078 ± 0.0037 | 4.25 | 0.0064 |
| ITGA9 | NM_002207 | 0.0752 ± 0.0098 | 0.3029 ± 0.0872 | 4.03 | 0.0004 |
| IL8R | NM_000634 | 0.0236 ± 0.0190 | 0.0925 ± 0.0754 | 3.92 | 0.0456 |
| IL1R | NM_000877 | 1.2685 ± 0.4128 | 4.8593 ± 2.1678 | 3.83 | 0.0027 |
| MAF-B | NM_005461 | 0.1294 ± 0.0559 | 0.4495 ± 0.2230 | 3.47 | 0.0196 |
| CCR10 | NM_016602 | 0.1192 ± 0.0608 | 0.4099 ± 0.3063 | 3.44 | 0.0524 |
| MAF | NM_005360 | 0.3977 ± 0.1476 | 1.3655 ± 0.6546 | 3.43 | 0.0063 |
| CSF1 | NM_000757 | 0.1021 ± 0.0364 | 0.3225 ± 0.1419 | 3.16 | 0.0046 |
| NOTCH1 | NM_017617 | 0.1687 ± 0.0765 | 0.5208 ± 0.3960 | 3.09 | 0.0765 |
| PROX1 | NM_002763 | 0.0035 ± 0.0004 | 0.0103 ± 0.0034 | 2.94 | 0.0025 |
| COUPTF2 | NM_021005 | 0.0089 ± 0.0036 | 0.0245 ± 0.0061 | 2.74 | 0.0004 |
| VEGFR3 | NM_002020 | 0.0072 ± 0.0071 | 0.0175 ± 0.0173 | 2.41 | 0.0869 |
| IFNγ | NM_000619 | 0.0693 ± 0.0150 | 0.1633 ± 0.0792 | 2.36 | 0.0346 |
| BCL6 | NM_001706 | 0.0232 ± 0.0099 | 0.0537 ± 0.0318 | 2.31 | 0.0356 |
| CXCR3 | NM_001504 | 1.3094 ± 0.6164 | 3.0218 ± 1.4978 | 2.31 | 0.0060 |
| NRP1 | NM_003873 | 0.0170 ± 0.0074 | 0.0362 ± 0.0139 | 2.13 | 0.0020 |
| C5aR1 | NM_000343 | 0.1807 ± 0.0684 | 0.3778 ± 0.2117 | 2.09 | 0.0348 |
| VEGFC | NM_005429 | 0.0001 ± 0.00003 | 0.0002 ± 0.00006 | 1.89 | 0.0679 |
| IL6R | NM_000565 | 0.0818 ± 0.0229 | 0.1497 ± 0.0232 | 1.83 | 0.0015 |
| NRP2 | NM_003872 | 0.0008 ± 0.0002 | 0.0014 ± 0.0005 | 1.81 | 0.0107 |
| TIE2 | NM_000459 | 0.0654 ± 0.0257 | 0.1106 ± 0.0684 | 1.69 | 0.0924 |
| IL10 | NM_000572 | 0.2299 ± 0.0766 | 0.3283 ± 0.1284 | 1.43 | 0.1091 |
| LYVE1 | NM_006691 | 0.0010 ± 0.0003 | 0.0014 ± 0.0001 | 1.43 | 0.0368 |
| PDPN | NM_006474 | 0.0003 ± 0.0001 | 0.0004 ± 0.0001 | 1.24 | 0.0211 |
| VEGFD | NM_004469 | 0.0024 ± 0.0009 | 0.0030 ± 0.0011 | 1.24 | 0.2155 |
| CSF1R | NM_005211 | 0.0164 ± 0.0066 | 0.0199 ± 0.0063 | 1.22 | 0.0333 |
| CDX2 | NM_001265 | 0.0275 ± 0.0087 | 0.0280 ± 0.0086 | 1.02 | 0.4726 |
| CD14 | NM_000591 | 0.0009 ± 0.0002 | 0.0009 ± 0.0012 | 0.97 | 0.7615 |
| TLR4 | NM_003266 | 0.0879 ± 0.0395 | 0.0620 ± 0.0369 | 0.70 | 0.0053 |
| c-KIT | NM_000222 | 1.3404 ± 0.7077 | 0.9434 ± 0.4486 | 0.70 | 0.0611 |
| CD33 | NM_001772 | 0.7858 ± 0.2958 | 0.2621 ± 0.1209 | 0.33 | 0.0074 |
