# Supplementary material for: Renal cell tumors convert natural killer cells to a proangiogenic phenotype
Source: Oncotarget. 2020 Jun 30;11(26):2571–85. doi: 10.18632/oncotarget.27654 (PMC7335666; doi:10.18632/oncotarget.27654)
Supplement: Supplementary file 3 [file oncotarget-11-2571-s003.docx]

**Supplementary Table 3: Gene expression results for RCC NK cells versus dNK cells**

| Gene | RCC pNK  mean^†^ | RCC TiNK  mean^†^ | TiNK/pNK Fold Change | *P*-value | dNK  Signal^‡^ | dNK Fold Change^#^ |
| --- | --- | --- | --- | --- | --- | --- |
| HIF1α | 0.1660 | 8.7794 | 52.88 | 0.0002 | 20 | 1 |
| CCL1 | 0.0216 | 0.8968 | 41.48 | 0.0023 | 20 | 1 |
| ANG1 | 0.0080 | 0.2912 | 36.44 | 0.0016 | 20 | 1 |
| IL6 | 0.0107 | 0.3577 | 33.57 | 0.0001 | 42 | 2.10 |
| VEGFA | 0.1108 | 3.6000 | 32.49 | 0.0019 | 135 | 6.75 |
| HIF2α | 0.0709 | 2.2735 | 32.05 | 0.0001 | 20 | 1 |
| IL7 | 0.0482 | 1.4989 | 31.12 | 0.0059 | 20 | 1 |
| ANG2 | 0.0774 | 2.1075 | 27.22 | 0.0057 | 46 | 2.30 |
| CXCL3 | 0.0828 | 2.1222 | 25.63 | 0.0284 | 34 | 1.70 |
| IL8 | 0.1673 | 4.1773 | 24.96 | 0.0004 | 94 | 4.70 |
| NFĸB2 | 0.0922 | 2.2475 | 24.36 | 0.0017 | 20 | 1 |
| CCL19 | 0.0838 | 1.9555 | 23.34 | 0.0041 | 20 | 1 |
| VEGFR1 | 0.0870 | 1.9562 | 22.48 | 0.0022 | 20 | 1 |
| CCL20 | 0.0298 | 0.5966 | 20.03 | 0.0114 | 20 | 1 |
| CCL3 | 0.0083 | 0.1593 | 19.14 | 0.0022 | 47 | 2.35 |
| TAL1 | 0.1242 | 2.3565 | 18.98 | 0.0060 | 31 | 1.55 |
| HOXA4 | 0.1923 | 3.1505 | 16.38 | 0.0037 | 36 | 1.80 |
| TNFα | 0.0221 | 0.3151 | 14.29 | 0.0010 | 20 | 1 |
| HOXA9 | 0.1090 | 1.5564 | 14.28 | 0.0027 | 20 | 1 |
| IL15 | 0.0117 | 0.1637 | 14.00 | 0.0510 | 20 | 1 |
| IL5 | 0.0563 | 0.7603 | 13.50 | 0.0003 | 20 | 1 |
| DLL4 | 0.0024 | 0.0321 | 13.42 | 0.0025 | 20 | 1 |
| NFĸB1 | 0.1735 | 2.2153 | 12.77 | 0.0009 | 20 | 1 |
| IL1β | 0.0188 | 0.2371 | 12.61 | 0.0003 | 20 | 1 |
| CXCL1 | 0.0586 | 0.7290 | 12.43 | 0.0008 | 147 | 7.35 |
| CD133 | 0.0047 | 0.0555 | 11.85 | 0.0013 | 20 | 1 |
| SIX1 | 0.0615 | 0.6290 | 10.22 | 0.0079 | 20 | 1 |
| REL-A | 0.0163 | 0.1587 | 9.72 | 0.0001 | 20 | 1 |
| PECAM1 | 0.0586 | 0.5631 | 9.61 | 0.0001 | 31 | 1.55 |
| CXCR1 | 0.1570 | 1.4546 | 9.27 | 0.0060 | 20 | 1 |
| CD34 | 0.1929 | 1.6451 | 8.53 | 0.0039 | 20 | 1 |
| CCR7 | 0.3483 | 2.9437 | 8.45 | 0.0329 | 107 | 5.35 |
| IL10R | 0.0207 | 0.1720 | 8.32 | 0.0055 | 20 | 1 |
| CXC3R1 | 0.2793 | 2.1685 | 7.76 | 0.0080 | 20 | 1 |
| IL4 | 0.0401 | 0.2809 | 7.01 | 0.0545 | 27 | 1.35 |
| CCR1 | 0.4108 | 2.8239 | 6.87 | 0.0063 | 20 | 1 |
| VEGFR2 | 0.3516 | 2.2583 | 6.42 | 0.0004 | 20 | 1 |
| BCL2 | 0.0454 | 0.2842 | 6.26 | 0.0229 | 20 | 1 |
| NFATc1 | 0.1118 | 0.6969 | 6.23 | 0.0010 | 20 | 1 |
| CCR3 | 0.6665 | 3.9615 | 5.94 | 0.0024 | 20 | 1 |
| VEGFB | 0.0472 | 0.2738 | 5.80 | 0.0000 | 91 | 4.55 |
| PAX6 | 0.0861 | 0.4903 | 5.70 | 0.0063 | 20 | 1 |
| IL3 | 0.0474 | 0.2423 | 5.11 | 0.0005 | 20 | 1 |
| PAX4 | 0.0012 | 0.0060 | 5.02 | 0.00001 | 20 | 1 |
| IL33 | 0.0506 | 0.2423 | 4.79 | 0.0110 | 20 | 1 |
| IL7R | 0.4150 | 1.9554 | 4.71 | 0.0186 | 20 | 1 |
| CD146 | 0.0054 | 0.0245 | 4.52 | 0.0079 | 1076 | 53.80 |
| IL3R | 0.0142 | 0.0610 | 4.30 | 0.0030 | 20 | 1 |
| TEAD2 | 0.0018 | 0.0078 | 4.25 | 0.0064 | 20 | 1 |
| ITGA9 | 0.0752 | 0.3029 | 4.03 | 0.0004 | 20 | 1 |
| IL8R | 0.0236 | 0.0925 | 3.92 | 0.0456 | 20 | 1 |
| IL1R | 1.2685 | 4.8593 | 3.83 | 0.0027 | 20 | 1 |
| MAF-B | 0.1294 | 0.4495 | 3.47 | 0.0196 | 20 | 1 |
| CCR10 | 0.1192 | 0.4099 | 3.44 | 0.0524 | 20 | 1 |
| MAF | 0.3977 | 1.3655 | 3.43 | 0.0063 | 20 | 1 |
| CSF1 | 0.1021 | 0.3225 | 3.16 | 0.0046 | 20 | 1 |
| NOTCH1 | 0.1687 | 0.5208 | 3.09 | 0.0765 | 20 | 1 |
| PROX1 | 0.0035 | 0.0103 | 2.94 | 0.0025 | 20 | 1 |
| COUPTF2 | 0.0089 | 0.0245 | 2.74 | 0.0004 | 29 | 1.45 |
| VEGFR3 | 0.0072 | 0.0175 | 2.41 | 0.0869 | 25 | 1.25 |
| IFNγ | 0.0693 | 0.1633 | 2.36 | 0.0346 | 120 | 6.00 |
| BCL6 | 0.0232 | 0.0537 | 2.31 | 0.0356 | 20 | 1 |
| CXCR3 | 1.3094 | 3.0218 | 2.31 | 0.0060 | 20 | 1 |
| NRP1 | 0.0170 | 0.0362 | 2.13 | 0.0020 | 20 | 1 |
| C5aR1 | 0.1807 | 0.3778 | 2.09 | 0.0348 | 20 | 1 |
| VEGFC | 0.0001 | 0.0002 | 1.89 | 0.0679 | 120 | 6.00 |
| IL6R | 0.0818 | 0.1497 | 1.83 | 0.0015 | 20 | 1 |
| NRP2 | 0.0008 | 0.0014 | 1.81 | 0.0107 | 20 | 1 |
| TIE2 | 0.0654 | 0.1106 | 1.69 | 0.0924 | 76 | 3.80 |
| IL10 | 0.2299 | 0.3283 | 1.43 | 0.1091 | 20 | 1 |
| LYVE-1 | 0.0010 | 0.0014 | 1.43 | 0.0368 | 20 | 1 |
| PDPN | 0.0003 | 0.0004 | 1.24 | 0.0211 | 20 | 1 |
| VEGF-D | 0.0024 | 0.0030 | 1.24 | 0.2155 | 20 | 1 |
| CSF1R | 0.0164 | 0.0199 | 1.22 | 0.0333 | 20 | 1 |
| CDX2 | 0.0275 | 0.0280 | 1.02 | 0.4726 | 20 | 1 |
| CD14 | 0.0009 | 0.0009 | 0.97 | 0.7615 | 20 | 1 |
| TLR4 | 0.0879 | 0.0620 | 0.70 | 0.0053 | 20 | 1 |
| c-KIT | 1.3404 | 0.9434 | 0.70 | 0.0611 | 28 | 1.4 |
| CD33 | 0.7858 | 0.2621 | 0.33 | 0.0074 | 20 | 1 |

† Gene expression determined by RT-qPCR

‡ Gene expression determined by microarray analysis on purified using CodeLinkTM Uniset Human 20K I Bioarray where minimum hybridization threshold equals 20 [see ref. 5].

# Calculated by dividing signal by minimum hybridization threshold.
